# Ethnopharmacological Survey of Herbal Remedies Used for the Treatment of Cancer in the Greater Casablanca-Morocco

**DOI:** 10.1155/2019/1613457

**Published:** 2019-07-21

**Authors:** Mohammed Bourhia, Abdelaaty Abdelaziz Shahat, Omer Mohammed Almarfadi, Fahd Ali Naser, Wael Mostafa Abdelmageed, Amal Ait Haj Said, Fatiha El Gueddari, Abderrahim Naamane, Laila Benbacer, Naima Khlil

**Affiliations:** ^1^Laboratory of Chemistry, Biochemistry Nutrition, and Environment, Faculty of Medicine and Pharmacy, University Hassan II, Casablanca, Morocco; ^2^Department of Pharmacognosy (Medicinal Aromatic and poisonous Plants Research Center), College of Pharmacy, King Saud University, P.O. Box 2457, Riyadh 11451, Saudi Arabia; ^3^Chemistry of Medicinal Plants Department, National Research Centre, 33 El-Bohouth St., P.O. Box 12622, Dokki, Giza, Egypt; ^4^Pharmacognosy Department, Faculty of Pharmacy, Assiut University, Assiut 71526, Egypt; ^5^Laboratory of Pharmacognosy, Faculty of Medicine and Pharmacy of Casablanca, Hassan II University, Casablanca, Morocco; ^6^Life Science Division. National Centre for Energy, Sciences, and Nuclear Techniques. B.P 1382 RP, 10001 Rabat, Morocco

## Abstract

Medicinal plants played an important role in traditional medicine for the treatment of diseases since antiquities. The aim of the study is to carry out an ethnobotanical survey on medicinal plants used traditionally in cancer treatment in the region of Greater Casablanca-Morocco, and to enhance the traditional herbal medicine knowledge. 110 traditional healers in the study area were interviewed face to face to response a survey questionnaire including the names of plants used in cancer treatment, used parts, methods of preparation, and route of administration. Indices of Fidelity Level (FL), Use Value (UV), and Relative Frequency of Citation (RFC) were calculated to identify the most effective plants used for cancer treatment. Eight species were specified including* Aristolochia baetica, Aristolochia paucinervis*,* Bryonia dioica*,* Aquilaria malaccensis, Marrubium vulgare, Lavandula maroccana, Ephedra alata,* and* Euphorbia resinifera* belonging to 7 families. Aristolochiaceae, Aquilariaceae, and Cucurbitaceae were the most useful families in cancer treatment with high significant indices of UV, FL, and RFC with values of 1, 100%, and 1, respectively. Roots and leaves were the most commonly used plant parts. Decoction and powder mixed with honey were the frequently used method for remedies preparation. The present study showed that the people living in Morocco's economic capital are still highly dependent on traditional herbal medicine for the treatment of cancer. Therefore, it seems that herbal medicine still plays a crucial role in the primary healthcare system for the local population. During this survey, it was reported that even some plant families are highly toxic for humans like Aristolochiaceae that are frequently used in cancer treatment in the study area. As many people still rely on natural traditional medicine considering (it is safe with no side effects), so combined effort between all social categories including scientists and traditional healers should be established for involving the scientific validity of the used plants in the treatment of diseases.

## 1. Introduction

The conventional utilization of medicinal plants for the treatment of different disease around the world is in practice since incident time with the development of civilization. Both animals and plants have gifted potential for the discovery of drugs [1, 2]. Medicinal plants are utilized for the healing of different infections and contributed as a foundation of motivation for novel therapeutic agents. 80% of the world still depend on the traditional uses of medicinal plants [3, 4]. Cancer is defined as a disease in which abnormal cells divide in an uncontrolled way within the body of aberrational forms of the body's own cells. All cancer types drain through a series of steps described by progressive loss of normal growth control. Cancer can affect people at all ages even fetus; however, the risk for most varieties increases with age [5]. Cancer has an enormous impact on the healthcare economy and represents a great health burden and exhausts healthcare resources worldwide. It is estimated that 7.6 million people died from cancer in the world in 2007 [6]. In Morocco, nearly 40,000 new cases are diagnosed with cancer each year [7]. About 70 to 80% of patients in Africa are treated by traditional medicine due to the high costs of conventional medicines; many other people still rely on traditional medicine considering herbal medicines that have no side effects because of their natural origins and are often considered as safe drugs [8].

In Morocco, more than 50% of the population live in rural areas where access to conventional healthcare facilities is rare. Moreover, this access is so difficult if the geographical location is mountainous. Taking into account these conditions, local people rely strongly on folkloric herbal medicine than current synthetized drugs [9]. Morocco is recognized for its ecological diversity due to its geographical location as one of the Mediterranean countries with a long history in medical tradition and ancestral know-how of folkloric herbal medicine [10]. The knowledge of using medicinal plants and the procedures applied to their preparation has been inherited from one generation to upcoming either verbally or in writing [11]. The traditional inheritance may be facing extinction if it is not transmitted to next generation [12], many other factors causing a rapid loss of this knowledge like alteration of physical and biological environments, expertise loss due to death of the elderly, migration, rural exodus, acculturation, and modernization [13]. Serval authors have studied the traditional pharmacopeia in Morocco, whereas very little or no information was listed on the medicinal plants used for cancer treatment [14].

Willing to contribute to the safeguarding of herbal traditional remedies knowledge and to make it easy to find, to use, and to be more familiarized with cancer treatment, the present work was conducted to highlight the medicinal plants used in the traditional preparation for cancer treatment. Used parts, methods of preparation, and route of administration were investigated.

## 2. Material and Methods

### 2.1. Study Area

Casablanca is the largest Moroccan city, located 90 km south of Moroccan capital (Rabat) stretched on the Atlantic Ocean and limited to Chaouia - Ourdigha region to the northeast (33° 31′ 59.99′′ N -7° 34′ 59.99′′ W). The city has grown from a small port at the beginning of the 20th century to one of the biggest African cities.

### 2.2. Economic Interest

Nowadays Casablanca is considered as the most economic and commercial capital of the Kingdom. Casablanca not only plays a key role in trading economics for the African-European region, but also is the strongest industrial centre of Morocco with more than half of the country's factories investment and half of Morocco's commercial banking transactions [15].

### 2.3. Surface

Greater Casablanca region is one of the sixteen Moroccan regions covering an area of 1140.54 km^2^. Actually, the urban area is the most densely populated with 227.82 km2 per square kilometers or 18.8%. In early 1980, the urban areas covered about 100.0 per square kilometers Casablanca region boasting a population of 3,359,818 people according to data from 2014. [16].

### 2.4. Climate

The climate conditions in Greater Casablanca are very important for vegetation diversity for numerous reasons. The climate of Greater Casablanca is characterized by as mild winter with average temperature of about 13°C, and dry summer is influenced by semiarid climates and maximum temperature of 38/40°C. The spring is described as mild weather in which the average temperature rises to 28/30°C, while the autumn is characterized by the return of Atlantic weather, in which the average temperature reaches 20°C [17].

### 2.5. Vegetation

In the Greater Casablanca region, vegetation is an essential aspect of land cover; the dominant plants in the study area are palms, cactuses, pines and Mediterranean sclerophyllous. Forests found naturally in Grand Casablanca region are characterized by high vegetal diversity and are attractive locations for collecting plants. More than three great forests cover Casablanca land. The forest of Oued Nfifikh located in the north of Casablanca and the east of Mohammedia city. Bouskoura forest is the biggest forest in the region situated nearly south of Casablanca city. Other forests are situated in the study area like Echellalat forests located at the western border of Oued El Maleh. The regions of Greater Casablanca especially those covered by mainly productive types of vegetation are irrigated croplands or grasslands on fertilized soils that reach the water from natural sources including rivers which play a crucial role in maintaining the vegetation sustainability in the study area [18].

### 2.6. Data Collection

**Figure 1:**
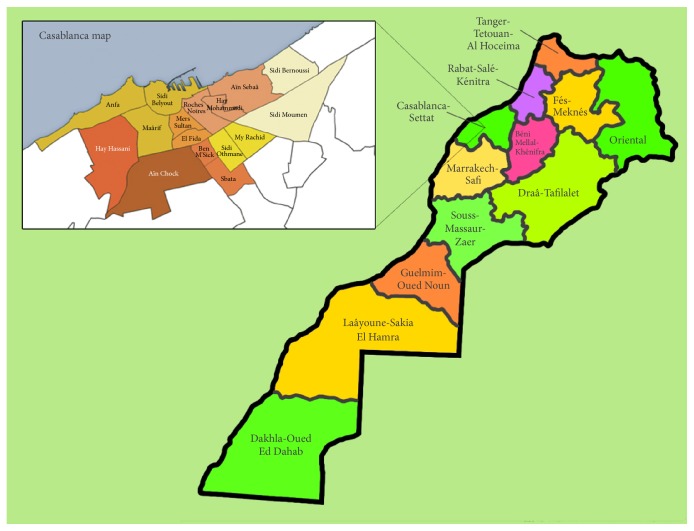
Map of the study area (Greater Casablanca location).

**Figure 2:**
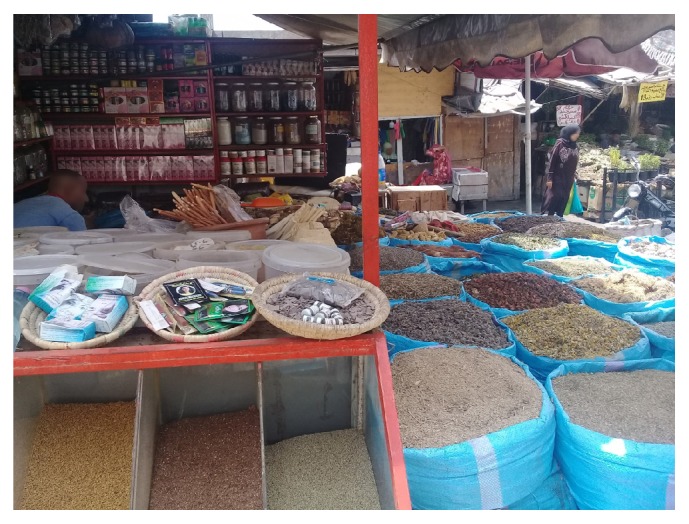
Shops of interviewed herbalists.

The ethnobotanical survey was conducted from December 2018 to April 2018. During this period different places throughout Casablanca city were visited (Figure 1). The study was effectuated by interviews with different herbalists and traditional healers who used herbal remedies in the treatment and prevention of cancer (Figure 2).

During the data collection, we used a survey questionnaire for explaining to herbalists and traditional healers the objectives of the study and the importance of providing the information in order to obtain their agreement to participate and to maintain transparency in the study. Data collection was carried out depending on the interviews following the survey questionnaire written for the circumstance. During the interview, 110 traditional herbalists were selected randomly throughout Casablanca city and were asked to answer a face-to-face questionnaire focused on the following points:

- Local names of the plants used in the treatment of cancer
- Parts of the plants used
- Routes of administration
- Preparation methods

The major collected plants during the survey were identified and voucher specimens have been deposited in the Herbarium of Scientific Institute of University Mohammed V–Rabat–Morocco under numbers* Aristolochia baetica* #101544,* Aristolochia paucinervis* #101545,* Bryonia dioica* #101547,* Aquilaria malaccensis *#101549*, Marrubium vulgare *#101551*, Lavandula maroccana*#101552*, Ephedra alata* #101553, and* Euphorbia resinifera *#101555

### 2.7. Statistical Analysis

#### 2.7.1. Fidelity Level (FL)

The collected data from the survey questionnaire were analyzed using a quantitative method including Fidelity Level (FL), Use Value (UV), and Relative Frequency of Citation (RFC). This method enables us to make a consensus of the plant species which is frequently used to treat cancer. FL indicates the percentage of informants pronounce to use plant species for the same purpose regarding diseases treatment. Fidelity level is calculated using the following Equation:

(1) $$\begin{array}{c} FL\left(\;\%\;\right)=\frac{N_{P}}{N}\times 100, \end{array}$$

where Np indicates the number of interviewed that claimed to use a plant species to treat a particular disease and N indicates the interviewed that used herbs as a medicine to cure any disease [19]

#### 2.7.2. Use Value (UV)

UV was calculated using the following Equation: UV=*Ʃ*U/n

where U indicates the number of species cited by each interviewee and n is the total number of informants [20].

#### 2.7.3. Relative Frequency of Citation (RFC)

RFC index was calculated according to the following Equation:

(2) $$\begin{array}{c} RFC=\frac{FC}{N}\;\left(0<RFC<1\right). \end{array}$$

where FC indicates the number of interviewed claimed to use species in the treatment and N is the total number of interviewees [19].

## 3. Results and Discussion

### 3.1. Demographic Characteristics of Interviewees

110 traditional healers from Greater Casablanca participated in the study. 10 informants were women and 100 were men. Their age ranges from 30 to 60 years. All the informants use herbal medicine alone in the treatment of cancer. It was reported that the people in the region depend on the traditional treatment of cancer including natural preparation for the following reasons:

- The accessibility and the very low cost of herbal treatment compared to conventional treatment taking into account the low income of Moroccan people.
- Some of who believe that the medicinal plants are more effective against cancer disease than synthesized drugs
- Other interviewees considered the medicinal plants have no side effects compared to conventional drugs.

It was reported that the women traditional healers have full knowledge regarding the herbs used in alternative medicine compared to men traditional healers. The women inherited traditional medicine knowledge from their parents and save it for the future generation. The frequency of using the medicinal plants in the treatment increased with age. For these reasons, the present study is in agreement with elsewhere reported in the literature [21]. It was reported that the percentage of people aged more than 50, 40, and 20 years use the herbs in the treatment of diseases with frequency values of 57%, 18%, and13%, respectively [12].

### 3.2. Medicinal Plants Used by the Local Population

During the survey period, we collected information on 8 plant species, among 7 families and 7 genera used in cancer treatment in the Greater Casablanca region as listed in Table 1.

**Figure 3:**
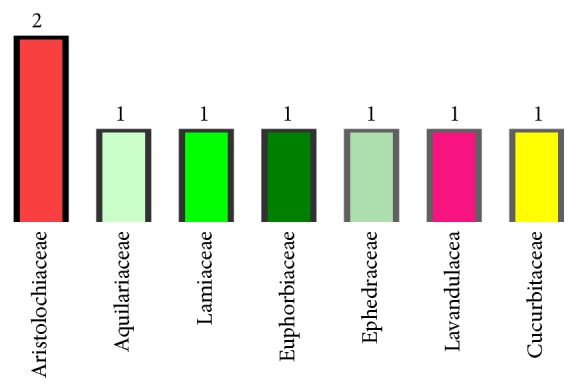
Number of medicinal species per botanical family.

The plant families reported in this inventory are Aristolochiaceae including two species, Aquilariaceae, Lamiaceae, Euphorbiaceae, Ephedraceae, Lavandulacea, and Cucurbitaceae with one species in each. Data are summarized in Figure 3.

It was reported that the most popular plant family used in Greater Casablanca for cancer treatment is Aristolochiaceae. The use of this family in cancer treatment is not limited to the studied area but it is also sold by traditional healers throughout all Moroccan cities for the same purpose of cancer treatment [22]. This ethnobotanical survey agrees with other scientific literature took place in North Africa which reported that the Aristolochiaceae belongs to the most effective family used in traditional treatment of cancer [23]. Lamiaceae is another family inventoried among the medicinal plants used in alternative medicine for cancer treatment [24]. It seems that the plant's families cited during the present survey are not used randomly in the traditional treatment of cancer. Therefore, several literatures have been seeking the scientific basis of using the mentioned plants families in this study, in which it was reported that Aristolochiaceae, Euphorbiaceae, and Aquilariaceae families have an interesting antitumor activity [25–27]. Lamiaceae, Cucurbitaceae, Ephedraceae, or their derivatives have been shown antiproliferative and cytotoxic effects against cancer cell lines [28–30].

All the medicinal herbs listed in the ethnobotanical survey carried out in the region of Greater Casablanca were wild species (Table 2). The results of the study showed a large vegetal diversity of plant species used in the traditional treatment of cancer in the study area. The knowledge depth on traditional uses of herbs in the treatment of diseases was reported as a key factor explaining the spreading of the enormous quantity of medicinal plants in the studied region for consideration for diseases treatment.

**Figure 4:**
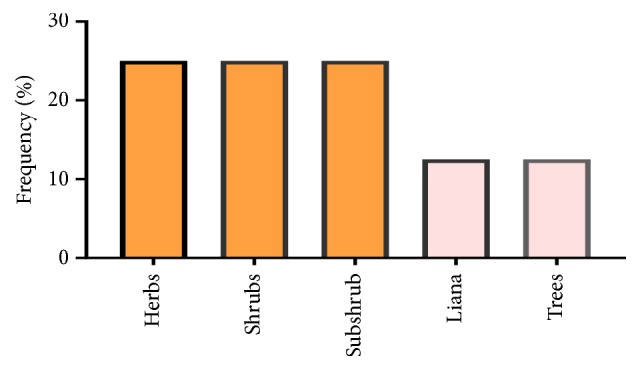
Growth forms (habits) of reported medicinal plant species used for the treatment of cancer in the Greater Casablanca.

The results of the biological analysis of plant form used in the treatment of cancer in the studied region showed the presence of Shrubs, subshrubs, and herbs with a percentage of 25 in each. Liana and trees were other biological forms listed with the percentage of 12.5 in each (Figure 4). The results of this study were used to perform a comparison [12], that resulted in that herbs' form was the most biologically effective form used in traditional treatment of disease in Morocco.

### 3.3. Ethnobotanical Indices

Fidelity Level (FL) indicates the choice by which the traditional healer uses a plant species for treatment a given disease. Use Value (UV) indicates the most cited plants. Relative Frequency of Citation (RFC) indicates the most desired plants for cancer treatment. Regarding these indices, the values of each species cited in the study are calculated from the available formula mentioned in the statistical section. FL and UV values of collected plants in the region of Greater Casablanca range from 10% to 100% and 0.07 to 1, respectively. RFC value ranges from 0.09 to 1 (Table 1). For screening the important medicinal herbs used in the region of Greater Casablanca for cancer treatment, we may analyze the obtained results of ethnobotanical indices of each plant species reported in the survey. 4 over 8 plant species such as* Aristolochia baetica, Aristolochia paucinervis, Bryonia dioica, *and* Aquilaria malaccensis* were cited with the highest values of FL, UV, and RFC (Table 1). Therefore the most useful plant species in the treatment of cancer are* Aristolochia baetica, Aristolochia paucinervis, Bryonia dioica, *and* Aquilaria malaccensis* with high RFC (1). The findings of our study were in consent with other studies showing the use of Aristolochia and Bryonia species in traditional treatment of cancer in Morocco [31].

It seems that, in the region of Greater Casablanca, a great conflict takes place between traditional healers regarding the plant collection and resources conservation. It was reported that many species in the region suffer extinction due to high collection conducted by the traditional healers. The results of the present study agree with another study [21], reporting that the collection pressure exercised on plants leads sometimes to uproot the whole plant instead of the required part. This collection manner combined with environmental factors can seriously compromise the sustainability of medicinal plants.

### 3.4. Plant Parts Used

**Figure 5:**
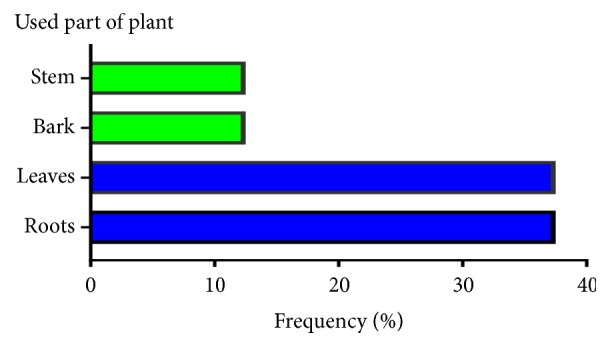
Frequency of plant parts used in natural preparation for cancer treatment.

Roots and leaves were the major plant parts used in cancer treatment with a percentage of 37.5 in each followed by stem and bark with a percentage of 12.5 in each (Figure 5).

The use of leaves in alternative medicine could be attributed to accessibility and the facility of collection than roots as mentioned in the literature [32]. Agreeing with our results, it was reported that the aerial parts are the most plant part used in the natural preparation for many years ago [33].

### 3.5. Preparation Method and Administration Route of Plant Drugs

**Figure 6:**
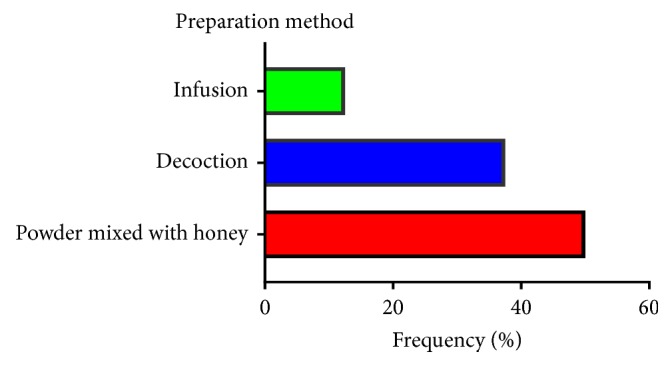
Frequency of preparation method of plant drugs.

50% of cited species including* Aquilaria malaccensis, Aristolochia baetica, Aristolochia paucinervis*, and* Bryonia dioica *are ground into a fine powder after drying at room temperature; a small quantity (around 1 gram ) of this powder is mixed with honey (around 1 gram) and sometimes with salted butter (around 0.5 gram), then prepared to be ingested orally. Sometimes the powder is mixed with tea for enhancing the oral administration. 37.5% of the listed species in the present study including* Euphorbia resiniferas, Ephedra alata, *and* Marrubium vulgare *are prepared for cancer treatment using decoction method. It was reported that 12.5% of the inventoried species including* Lavandula maroccana *are prepared by the infusion method. Sometimes the same plant could be administered in different preparation forms. The prepared remedies are always taken orally for cancer treatment (Figure 6).

### 3.6. Probable Mechanism of Actions of Plants Involved in Cancer Treatment

Dysregulation of apoptosis is still one of the current ways involved in cancer treatment [34]. Control of cancerous cell growing still depends on the ability of these cells to undergo apoptosis [35]. The most studied mechanism of actions of plants involved in cancer treatment as reported in earlier reports was the apoptosis through the mitochondrial intrinsic pathway as shown in the effect induced by aqueous extract of* A. longa *and* Bryonia dioica* on BL41 cancer cell lines [36]

### 3.7. Harmful Effects of Inventoried Plants

**Figure 7:**
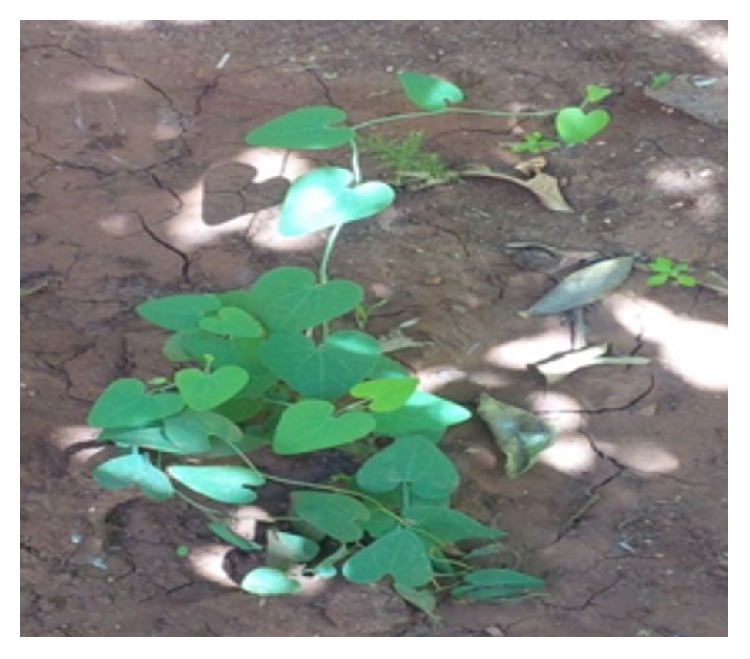
*Aristolochia baetica*.

**Figure 8:**
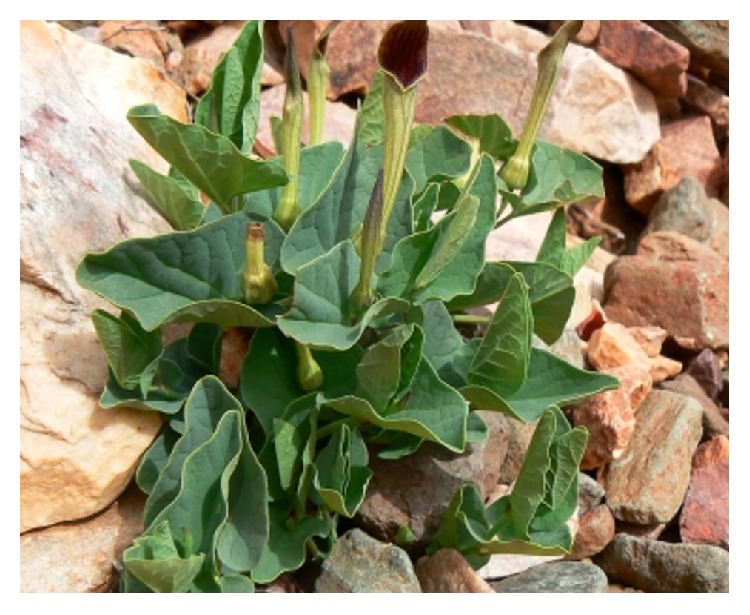
*Aristolochia paucinervis*.

**Figure 9:**
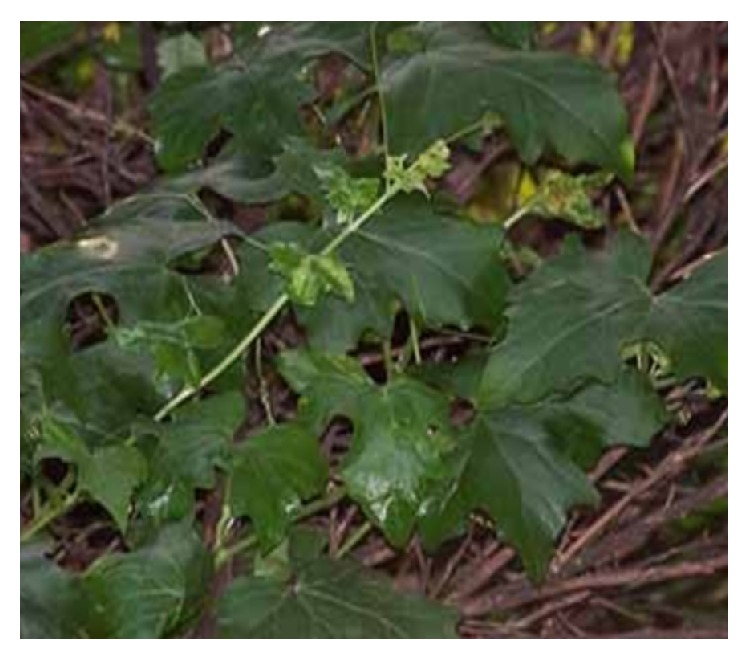
*Bryonia dioica*.

**Figure 10:**
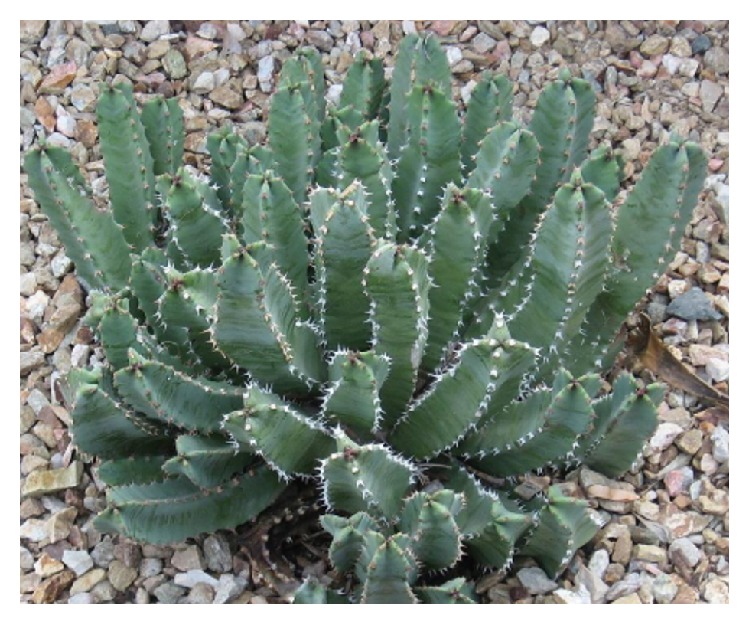
*Euphorbia resinifera*.

**Figure 11:**
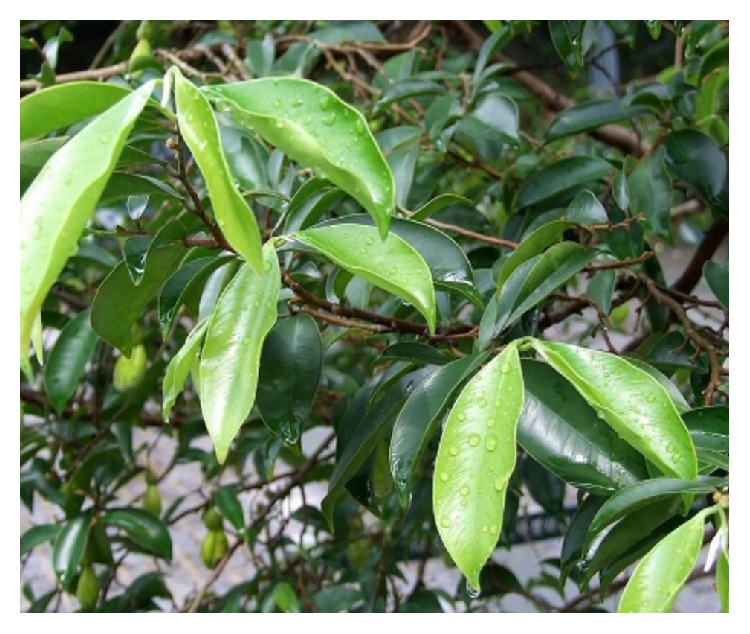
*Aquilaria malaccensis*.

**Figure 12:**
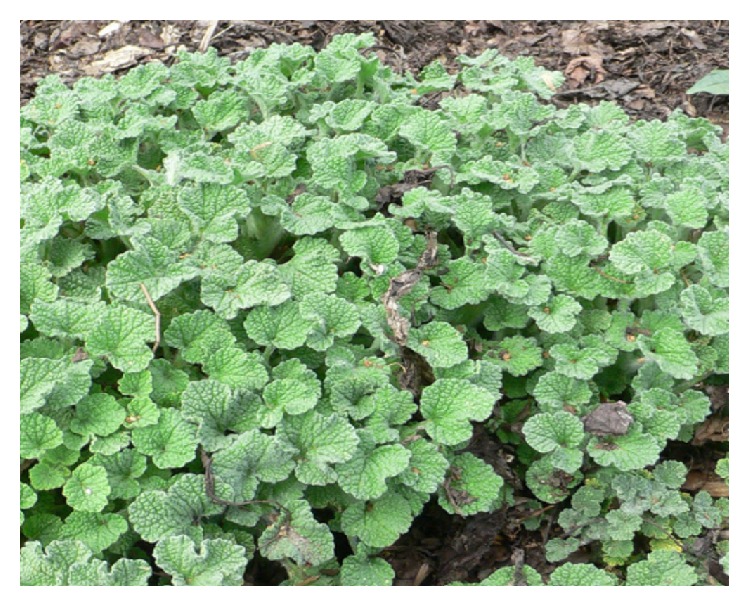
*Marrubium vulgare*.

**Figure 13:**
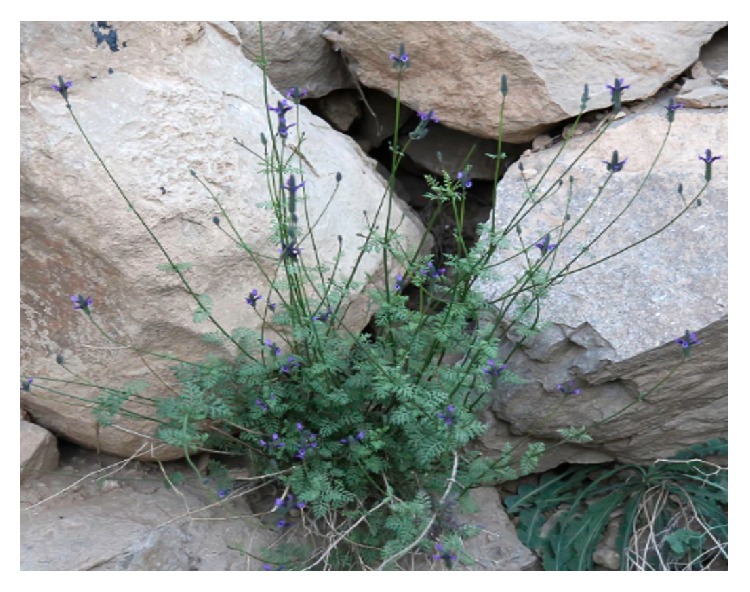
*Lavandula maroccana*.

**Figure 14:**
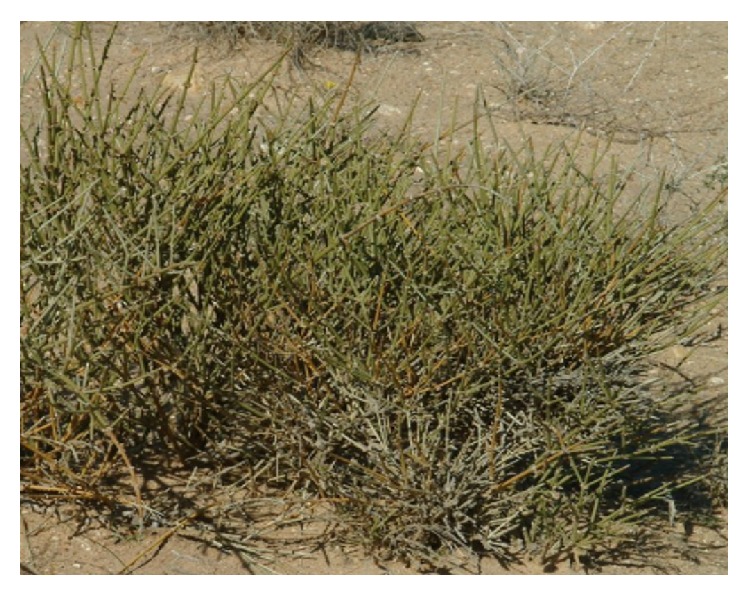
*Ephedra alata*.

*Aristolochia baetica *(Figure 7) and Aristolochia* paucinervis (*Figure 8), used for cancer treatment in the region of Greater Casablanca, were the most toxic plants [22, 37]. The toxic effects of these medicinal plants or their derivatives like aristolochic acids were largely investigated in several literatures [38]. It was reported that Aristolochia species induced renal failure and affected negatively the biochemical parameters in mice exposed to conditions of subacute toxicity.* Bryonia dioica *(Figure 9) is known for its acute toxicity, as reported in the literature, the LD_50_ of* Bryonia dioica *roots estimated at 340 mg/kg with oral administration to mice [39].* Euphorbia resinifera *(Figure 10) calcified belongs to the toxic plant also; it was reported that exposure to its latex induces oral, dermal, and ocular symptoms. This species was also having some irritant reaction with small concentration [40]. For acute and subacute toxicity study of* Aquilaria malaccensis *(Figure 11), no toxic effect was detected in treated mice with 2000mg/kg [41]. Regarding the other cited plants in the present work such as* Marrubium vulgare *(Figure 12),* Lavandula maroccana *(Figure 13), and* Ephedra alata* (Figure 14), no big data were available on their toxicities.

## 4. Conclusion

The present study provided data on the most popular medicinal plants used in cancer treatment in Greater Casablanca. Used parts, methods of preparation, and administration routes were also investigated. It also identified the knowledge serving anticancer herbs of local origin. It seems that some plant species listed in the survey have not been used randomly in cancer treatment, but scientifically they represented antiproliferative effects on cancer cell lines as reported in the literature. As some medicinal plants exhibiting a high toxic effect on humans were also used in remedies for cancer treatment, it became very important to pay more attention to study the medicinal plants safety.

## Figures and Tables

**Table 1:** Plants used for cancer treatment in the Greater Casablanca region.

Plant family	Plant species	Vernacular name	Used parts	Preparation method	Administration route	UV	FL	RFC
Aristolochiaceae	*Aristolochia baetica Aristolochia paucinervis*	bertzem khal	roots	powder mixed with honey	oral	1	100	1
Aquilariaceae	*Aquilaria malaccensis*	ighris	bark	powder mixed with honey	oral	1	100	1
Lamiaceae	*Marrubium vulgare*	meroute	leaves	decoction	oral	0.5	40	0.18
Euphorbiaceae	*Ephedra alata*	daghmous	leaves	decoction	oral	0.15	35	0.15
Ephedraceae	*Ephedra alata*	elaalnda	stem	decoction	oral	0.11	20	0.09
Lavandulacea	*Lavandula maroccana*	kouhila	leafy stem	infusion	oral	0.07	10	0.09
Cucurbitaceae	*Bryonia dioica*	berztem byad	roots	powder mixed with honey	oral	1	100	1

**Table 2:** Type of plants and habit.

Plant family	Plant species	Type of plants	Hbits
Aristolochiaceae	*Aristolochia baetica Aristolochia paucinervis*	Spontaneous	Shrub
Aquilariaceae	*Aquilaria malaccensis*	Spontaneous	Trees
Lamiaceae	*Marrubium vulgare*	Spontaneous	Herbs
Euphorbiaceae	*Ephedra alata*	Spontaneous	Subshrub
Ephedraceae	*Ephedra alata*	Spontaneous	Subshrub
Lavandulacea	*Lavandula maroccana*	Spontaneous	Herbs
Cucurbitaceae	*Bryonia dioica*	Spontaneous	Liana

## Data Availability

The data used to support the findings of this study are included within the article.
